# Ganetespib targets multiple levels of the receptor tyrosine kinase signaling cascade and preferentially inhibits ErbB2-overexpressing breast cancer cells

**DOI:** 10.1038/s41598-018-25284-0

**Published:** 2018-05-01

**Authors:** Harry Lee, Nipun Saini, Erin W. Howard, Amanda B. Parris, Zhikun Ma, Qingxia Zhao, Ming Zhao, Bolin Liu, Susan M. Edgerton, Ann D. Thor, Xiaohe Yang

**Affiliations:** 1 Julius L. Chambers Biomedical/Biotechnology Research Institute, Department of Biological and Biomedical Sciences, North Carolina Central University, North Carolina Research Campus, Kannapolis, North Carolina USA; 2 Department of Pathology, School of Medicine, University of Colorado Anschutz Medical Campus, Aurora, Colorado USA

## Abstract

Although ErbB2-targeted therapeutics have significantly improved ErbB2^+^ breast cancer patient outcomes, therapeutic resistance remains a significant challenge. Therefore, the development of novel ErbB2-targeting strategies is necessary. Importantly, ErbB2 is a sensitive client protein of heat shock protein 90 (HSP90), which regulates client protein folding, maturation, and stabilization. HSP90 inhibition provides an alternative therapeutic strategy for ErbB2-targeted degradation. In particular, ganetespib, a novel HSP90 inhibitor, is a promising agent for ErbB2^+^ cancers. Nevertheless, the anti-cancer efficacy and clinical application of ganetespib for ErbB2^+^ breast cancer is largely unknown. In our study, we examined the anti-cancer effects of ganetespib on ErbB2^+^ BT474 and SKBR3 breast cancer cells, and isogenic paired cancer cell lines with lentivirus-mediated ErbB2 overexpression. Ganetespib potently inhibited cell proliferation, cell cycle progression, survival, and activation/phosphorylation of ErbB2 and key downstream effectors in ErbB2^+^ breast cancer cells. Moreover, ganetespib decreased the total protein levels of HSP90 client proteins and reduced ErbB2 protein half-life. ErbB2-overexpressing cancer cells were also more sensitive to ganetespib-mediated growth inhibition than parental cells. Ganetespib also strikingly potentiated the inhibitory effects of lapatinib in BT474 and SKBR3 cells. Ultimately, our results support the application of ganetespib-mediated HSP90 inhibition as a promising therapeutic strategy for ErbB2^+^ breast cancer.

## Introduction

ErbB2 (Her2/Neu) is a member of the epidermal growth factor receptor (EGFR) family of receptor tyrosine kinases (RTKs). It is a preferential dimerization partner of other EGFR family members because of its high catalytic activity. Heterodimerization of ErbB2 with EGFR/ErbB1 or ErbB3 mediates downstream signaling pathways, including the PI3K/Akt/mTOR, Ras/Raf/MAPK/Erk, and Stat3 pathways, via autophosphorylation of the cytoplasmic tyrosine kinase domains of the receptors^1–3^. Aberrant ErbB2-mediated signaling can have oncogenic consequences, including increased cell proliferation, survival, and angiogenesis. As such, ErbB2 is amplified/overexpressed in up to 30% of invasive breast cancers and is associated with an aggressive phenotype, poor prognosis, and reduced overall patient survival^4–6^. In particular, ErbB2 is a validated therapeutic target for ErbB2-overexpressing breast cancers. The development and clinical application of ErbB2-targeted therapeutics, such as trastuzumab and lapatinib, have significantly improved clinical outcomes in patients with ErbB2-positive (ErbB2^+^) breast cancer. However, resistance, either primary or acquired, to these therapies is emerging as a significant challenge. Therefore, the development of novel therapeutics that may be complimentary to ErbB2-targeted therapies is of pivotal significance.

Heat shock protein 90 (HSP90) is a chaperone protein that regulates the folding, maturation, and stabilization of client proteins (e.g. ErbB2, ErbB3, Akt) that are involved in important cellular functions, such as proliferation, differentiation, and survival^7,8^. Overexpression/activation of HSP90 has been associated with the development of several human cancers, including breast cancer, and is associated with the stabilization of critical oncoproteins^9–11^. In particular, ErbB2 is a critical HSP90 client protein as it has been demonstrated that HSP90 interacts with the extracellular domain of ErbB2 and regulates its heterodimerization and activation to mediate downstream signaling pathways, such as PI3K/Akt and MAPK/Erk pathways^12,13^. In mouse and human models of ErbB2^+^ breast cancer, ErbB2 overexpression was shown to activate heat shock factor-1 (HSF1), a master transcription factor required for HSP90 synthesis, and stabilize various tumor-promoting HSP90 clients, including macrophage-migration inhibitory factor (MIF)^14^, Akt, EGFR, ErbB2, c-Raf, and mutated p53^15^. Also, in human breast cancer tissues, an increase in HSP90 levels was associated with an increase in cyclin D1, suggesting the role of HSP90 in cell proliferation and oncogenesis^16^. Similarly, HSP90 knockdown via RNAi in breast cancer cell and xenograft models induced cell cycle arrest at G1/S phase and downregulated Akt and NF-κB signaling^17^, which suggests the potential anti-cancer role of HSP90 inhibition. In regards to the clinical significance of HSP90 in ErbB2-mediated breast cancer, targeting HSP90 is emerging as a novel therapeutic strategy to destabilize and degrade its client proteins, particularly ErbB2.

Inhibition of HSP90 destabilizes and degrades its client proteins via the recruitment of E3 ubiquitin ligases, such as CHIP and Cullin-5^18–20^. In turn, proteasomal degradation of ErbB2 and other apical HSP90 client proteins further blocks downstream signaling pathways. Thus, targeted HSP90 inhibitors have been developed and tested as chemotherapeutic strategies for ErbB2^+^ cancers^21^. In particular, ganetespib (STA-9090) is a promising resorcinol-based HSP90 inhibitor with a unique triazolone moiety. In contrast to the first generation of geldanamycin-based HSP90 inhibitors, ganetespib has improved solubility and reduced risk of cardiac, ocular, and liver toxicities^22–25^. In preclinical models, ganetespib has shown significant anti-tumor effects in various solid tumors and hematologic malignancies by inhibiting cell proliferation via the induction of G2/M phase cell cycle arrest and apoptosis^24,26–30^. Particularly, Shimamura *et al*.^29^ reported that ganetespib induced the degradation of HSP90 client proteins, including both wild-type and mutant forms of EGFR, ErbB2, and KRAS, the induction of the pro-apoptotic factor Bim, and the downregulation of PI3K/Akt/mTOR and Raf/MEK/Erk signaling pathways in cell line and xenograft models of non-small cell lung cancer (NSCLC)^29^. Furthermore, these anti-cancer activities of ganetespib were initiated by the disruption of the HSP90-p23 chaperone complex in NSCLC cells^29^. In combination with other drugs, like taxanes, ganetespib significantly inhibited tumor growth in cell and xenograft models of NSCLC, triple-negative breast cancer (TNBC), and ovarian cancer^31–34^. Currently, various Phase I-III clinical trials are underway testing ganetespib alone or in combination with other drugs to treat NSCLC, breast, ovarian, and other advanced adenocarcinomas^25,35–38^. Although ErbB2 is a very sensitive HSP90 client protein, studies investigating the effects of ganetespib on ErbB2^+^ breast cancer are limited. In particular, ganetespib offers a promising chemotherapeutic strategy for refractory ErbB2^+^ breast cancers, even in the presence of mutations in the extracellular and tyrosine kinase domains of ErbB2, which are associated with primary resistance to ErbB2-targeting therapeutics. Nevertheless, the clinical significance and anti-cancer efficacy of ganetespib on ErbB2^+^ breast cancer is generally unknown and warrants further investigation.

Previously, we reported that ganetespib has anti-cancer effects on ErbB2^+^ gastric cancer cells^28^. Consistently, our data in the current study demonstrated that ganetespib significantly blocks oncogenic cellular processes, including cell proliferation, cell cycle progression, cell survival, and RTK signaling, and potentiates the anti-cancer effects of lapatinib in ErbB2^+^ BT474 and SKBR3 breast cancer cells. Collectively, our *in vitro* data provide critical evidence suggesting the potential clinical application of ganetespib as a therapeutic strategy for ErbB2^+^ breast cancer.

## Results

### Ganetespib inhibits cell proliferation in ErbB2^+^ breast cancer cells

**Figure 1:**
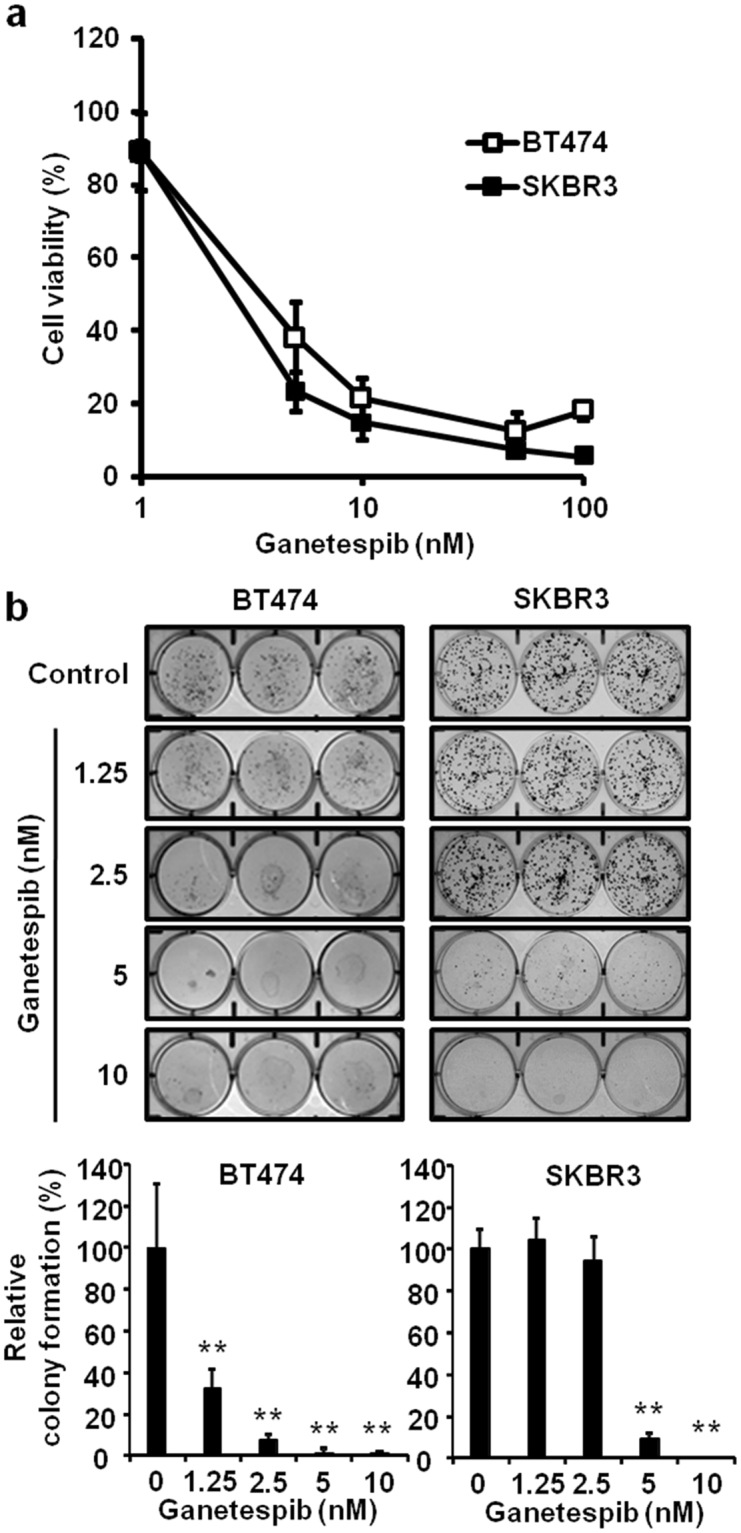
Ganetespib inhibits cell proliferation in ErbB2^+^ breast cancer cells. (**a**) BT474 and SKBR3 cells were treated with ganetespib (0, 1, 5, 10, 50, or 100 nM) and for 5 days, followed by an MTT assay to assess cell viability. The average percentages of viable cells relative to the untreated control cells in each cell line are graphed. (**b**) For the clonogenic assay, BT474, and SKBR3 cells were treated with ganetespib (0, 1.25, 2.5, 5, or 10 nM) for 21 and 14 days, respectively. Then, colonies were stained with crystal violet and quantified. Representative images of the crystal violet-stained colonies are shown in the top panels with the corresponding graphs in the lower panels. All values are presented as the means ± S.E. (*P ≤ 0.05, **P ≤ 0.01).

Previous studies indicate that ganetespib has anti-proliferative effects on ErbB2^+^ gastric cancer cells^27,28^. However, studies reporting the cellular responses to ganetespib in ErbB2^+^ breast cancer cells are limited. Therefore, we investigated the effects of ganetespib on cell proliferation/viability in BT474 and SKBR3 ErbB2^+^ breast cancer cell lines using an MTT (3-(4, 5-dimethylthiazol-2-yl)-2,5-diphenyltetrazolium bromide) assay. After ganetespib treatment (0–100 nM) for 5 days, cell growth was dose-dependently inhibited in both cell lines (Fig. 1a). As an additional measurement of ganetespib-mediated changes in cellular growth potential, we treated BT474 and SKBR3 cells with ganetespib (0–10 nM), followed by colony formation for 21 and 14 days, respectively. As shown in Fig. 1b, 1.25 nM and 5 nM of ganetespib significantly inhibited the colony-forming ability of BT474 and SKBR3 cells, respectively. Together, these data indicate that ErbB2^+^ breast cancer cells are sensitive to the anti-proliferative actions of ganetespib.

### Ganetespib induces G2/M cell cycle arrest in ErbB2^+^ breast cancer cells

**Figure 2:**
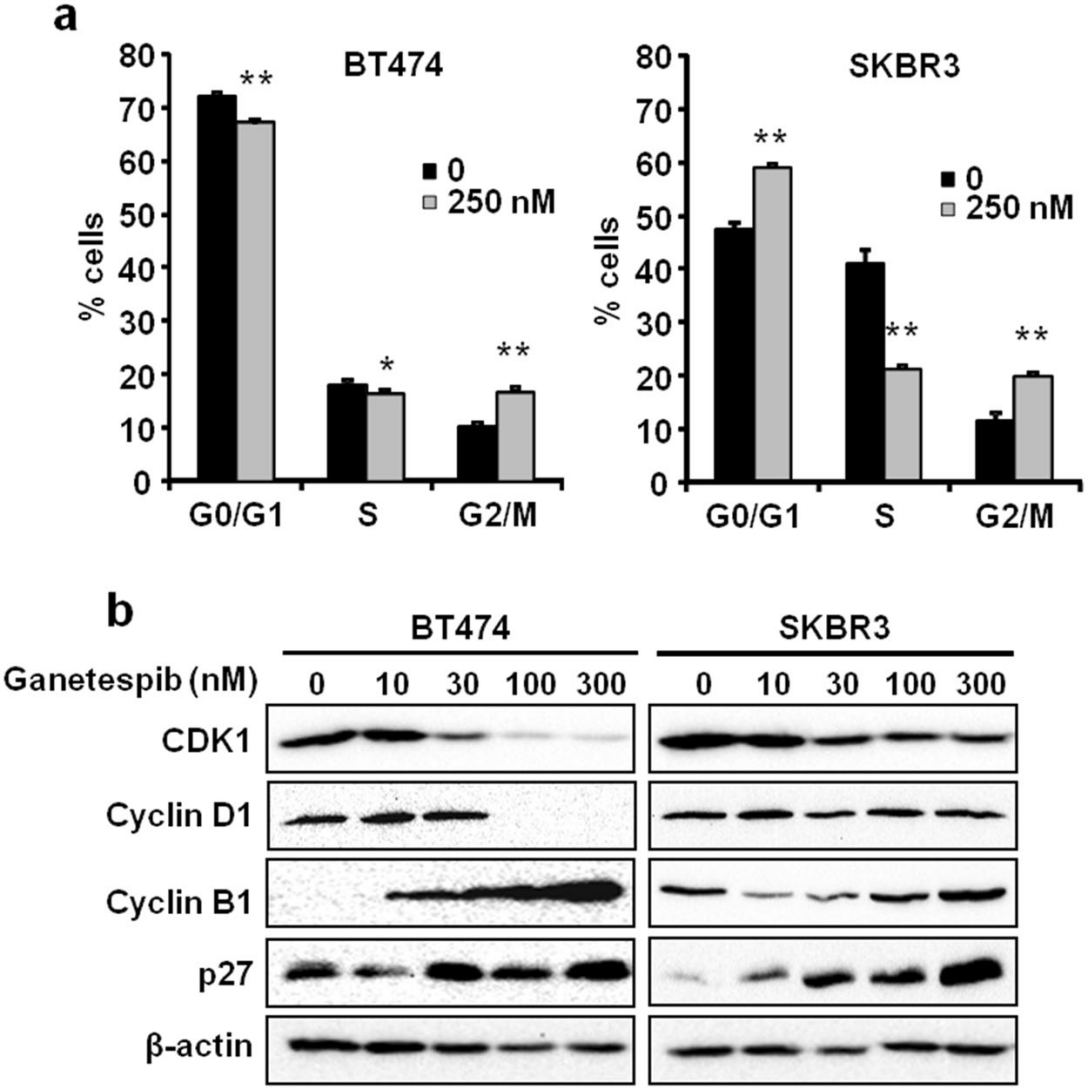
Ganetespib induces G2/M cell cycle arrest in ErbB2^+^ breast cancer cells. (**a**) BT474 and SKBR3 cells were treated with ganetespib (0 or 250 nM) for 24 hours, PI-stained, and analyzed for cell cycle distribution using flow cytometry. (**b)** Cropped Western blot images of the indicated cell cycle regulators are shown in ganetespib-treated (0–300 nM for 16 hours) BT474 and SKBR3 cells. All values are graphed as the means ± S.E. (*P ≤ 0.05; **P ≤ 0.01 as compared to the corresponding untreated controls).

Previous reports indicate that cell cycle arrest in the G2/M phase is a hallmark of ganetespib-mediated anti-proliferative effects in various cancer models, including gastric, thyroid, and small cell lung cancer^26–28,39^. In order to gain detailed insight into ganetespib-mediated growth inhibition, particularly in ErbB2^+^ breast cancer cells, we treated BT474 and SKBR3 cells with ganetespib (250 nM) for 24 hours, followed by cell cycle analysis using flow cytometry. As shown in Fig. 2a, ganetespib significantly induced G2/M phase arrest with a concurrent decrease in the percentage of proliferating cells in S phase in both cell lines. To note, ganetespib also markedly induced G0/G1 arrest only in the SKBR3 cells, which suggests cell line-specific cell cycle regulation mediated by ganetespib. These results showing that ganetespib stimulates the accumulation of ErbB2^+^ breast cancer cells in G2/M phase of the cell cycle are consistent with previous reports indicating the anti-proliferative effects of ganetespib in various cancer models.

Given the multitude of cell cycle regulators that are necessary for physiological cell proliferation, we investigated the effects of ganetespib on key cell cycle regulators involved in G0/G1 and G2/M checkpoints. To this end, BT474 and SKBR3 cells were treated with ganetespib (0-300 nM) for 16 hours. Western blot analysis revealed that ganetespib dose-dependently suppressed Cyclin-dependent kinase 1 (CDK1) and increased Cyclin B1 and p27 in both cell lines (Fig. 2b). At higher concentrations of ganetespib, Cyclin D1 expression was also drastically reduced, but only in the BT474 cells, further indicating cell line-specific differences in ganetespib-mediated cell cycle regulation. Overall, our data suggest that ganetespib modulates cell cycle regulators to elicit anti-proliferative effects in ErbB2^+^ breast cancer cells.

### Ganetespib induces apoptosis in ErbB2^+^ breast cancer cells

**Figure 3:**
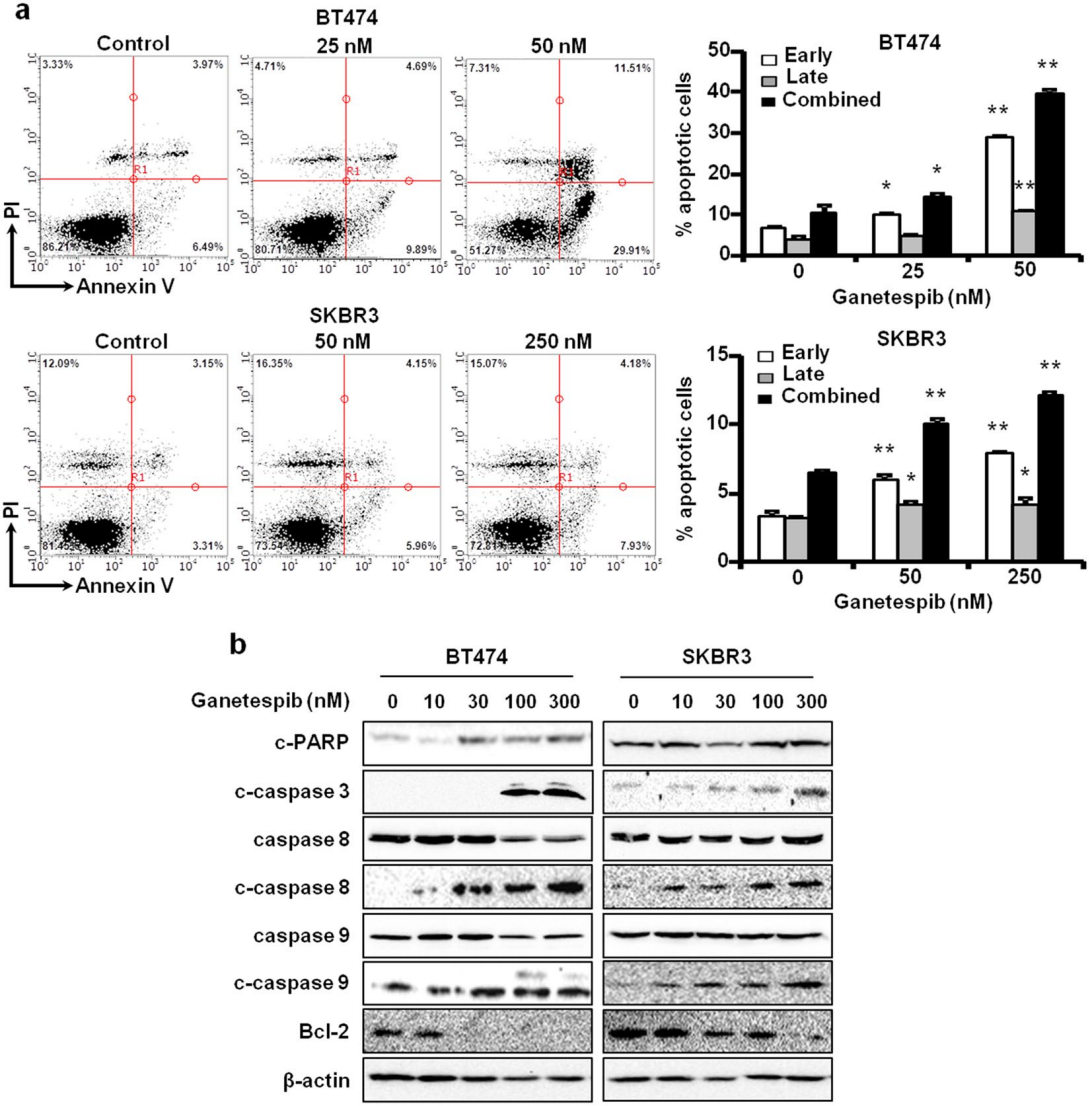
Ganetespib induces apoptosis in ErbB2^+^ breast cancer cells. (**a**) BT474 and SKBR3 cells were treated with ganetespib (25–250 nM) for 48 hours. Then, cells were Annexin V/PI-stained and analyzed with flow cytometry. The percentages of cells in early (Annexin V^+^/PI^−^) and late (Annexin V^+^/PI^+^) stages of apoptosis are graphed in the panels to the right of the representative dot plots. (**b**) Cropped images of Western blots at the target molecular weights for the indicated apoptotic markers are shown in ganetespib-treated (0, 10, 30, 100, 300 nM for 16 hours) BT474 and SKBR3 cells. All values are graphed as the means ± S.E. (*P ≤ 0.05; **P ≤ 0.01 as compared to the corresponding untreated controls).

The induction of apoptosis is regarded as an important anti-cancer mechanism of ganetespib in various cancer models^24,28,29^. Since ganetespib-mediated effects on apoptosis in ErbB2^+^ breast cancer cells have not been well-documented, we treated BT474 and SKBR3 cells with ganetespib (0-250 nM) for 16 hours, followed by Annexin V and propidium iodide (PI) staining to examine the effects of ganetespib on apoptosis. Based on flow cytometry analysis of Annexin V and PI-stained cells, we determined that ganetespib induced a dose-dependent increase in the percentages of cells in early (Annexin V^+^/PI^−^) and late (Annexin V^+^/PI^+^) stages of apoptosis (Fig. 3a). In particular, BT474 cells appeared to be more sensitive to ganetespib-induced apoptosis than the SKBR3 cells, as demonstrated by the drastic induction of early stage apoptosis at 50 nM ganetespib. Consistent with previous reports in other cancer models, our data provide evidence that ganetespib promotes apoptosis in ErbB2^+^ breast cancer cells.

To further uncover the mechanism of ganetespib-induced apoptosis in ErbB2^+^ breast cancer cells, we analyzed the expression and/or activation of key apoptotic markers involved in death receptor- and mitochondria-mediated pathways of cell death, after ganetespib treatment. As such, ganetespib (0-300 nM for 16 hours) induced PARP, caspase 3, caspase 8, and caspase 9 cleavage in a dose-dependent manner in BT474 and SKBR3 cells (Fig. 3b), which corroborates the ganetespib-mediated accumulation of apoptotic cells shown in Fig. 3a. Additionally, Bcl-2, an apoptotic antagonist, was decreased by ganetespib in both cell lines. Taken together, ganetespib-induced apoptosis is mediated by the induction of death receptor and mitochondrial pathways in ErbB2^+^ breast cancer cells.

### Ganetespib inhibits RTK signaling in ErbB2^+^ breast cancer cells

**Figure 4:**
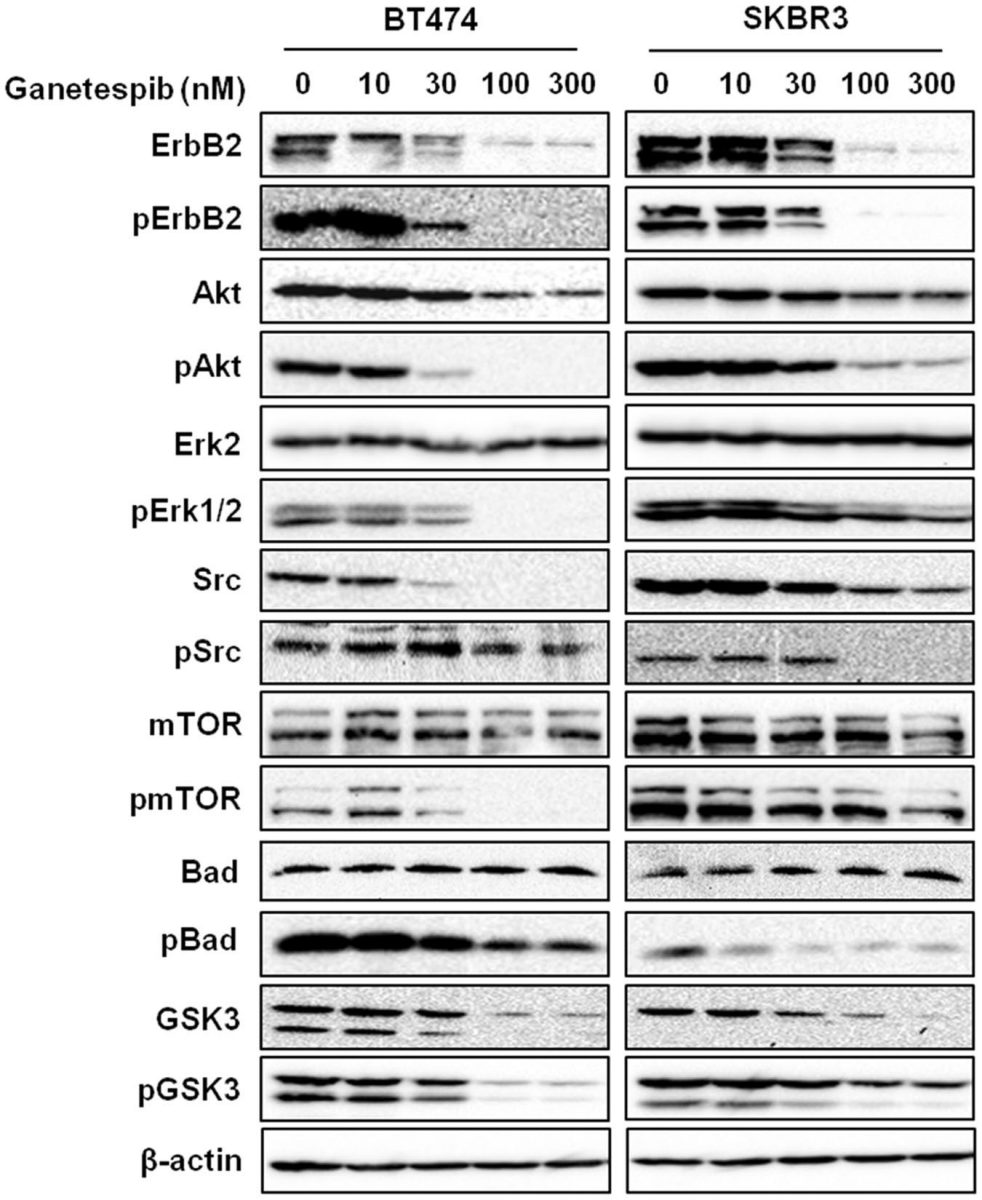
Ganetespib inhibits RTK signaling in ErbB2^+^ breast cancer cells. BT474 and SKBR3 cells were treated with ganetespib (0, 10, 30, 100, or 300 nM) for 16 hours, followed by Western blot analysis (cropped images) of the expression and activation/phosphorylation of the indicated markers involved in RTK signaling.

To further understand the molecular mechanisms of ganetespib-mediated inhibition of ErbB2^+^ breast cancer cells, the effects of ganetespib on oncogenic RTK signaling were investigated using Western blot analysis. As shown in Fig. 4, ganetespib dose-dependently decreased the activation of ErbB2, Akt, Erk1/2, Src, mTOR, Bad, and GSK3, indicating ganetespib-induced effects on apical and effector signaling molecules in the RTK signaling cascade. Importantly, ganetespib also promoted a dose-dependent decrease in the levels of total ErbB2, Akt, Src, and GSK3, which are putative client proteins of HSP90^21,40^. Our findings corroborate the inhibition of oncogenic RTK signaling elicited by ganetespib in ErbB2^+^ breast cancer cells^41^, which further supports the multi-faceted anti-cancer potential of ganetespib.

### Ganetespib decreases the protein half-life of ErbB2 in ErbB2^+^ breast cancer cells

**Figure 5:**
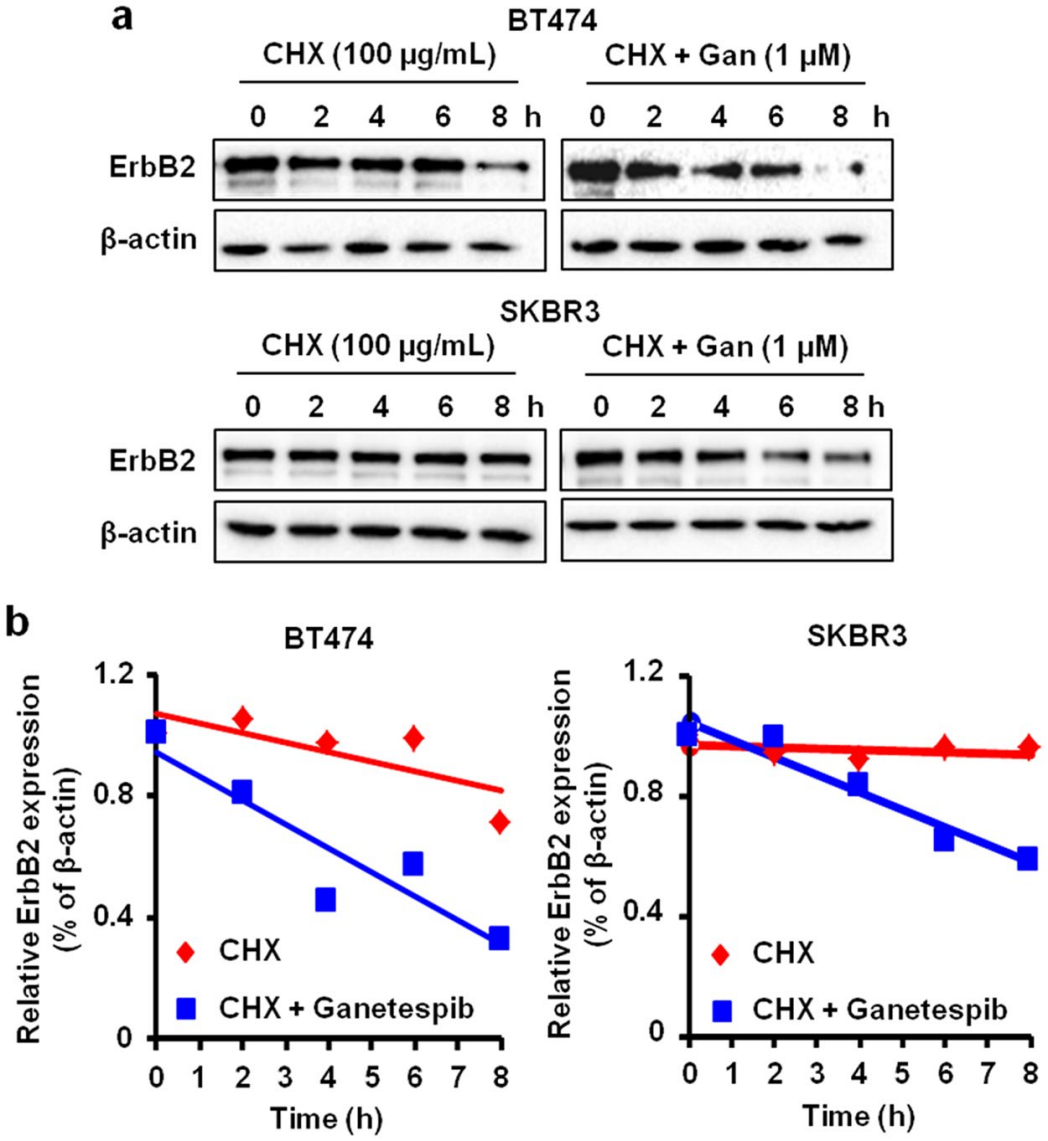
Ganetespib decreases the protein stability and half-life of ErbB2 in ErbB2^+^ breast cancer cells. (**a**) BT474 and SKBR3 cells were treated with CHX (100 μg/mL) +/− ganetespib (1 μM) for the indicated treatment durations. Then, ErbB2 protein expression was analyzed using Western blotting. Cropped images of ErbB2 and β-actin protein expression are shown. (**b**) ErbB2 protein band intensities were quantified using ImageJ software and normalized to corresponding β-actin protein band intensities. Relative ErbB2 protein expression after CHX +/− ganetespib treatments for each cell line is graphed.

HSP90 acts as a chaperone protein to maintain the stability of its client proteins. In turn, client protein degradation via the inhibition of HSP90 is a major regulatory mechanism of ganetespib^7,8^. As such, the dose-dependent decrease in total ErbB2, Akt, Src, and GSK3 expression after ganetespib treatment in the ErbB2^+^ breast cancer cells (Fig. 4) is indicative of potential protein degradation. To examine the specific effects of ganetespib on ErbB2 protein stability in the ErbB2^+^ breast cancer cells, we treated BT474 and SKBR3 cells with cycloheximide (CHX; 100 µg/mL), an inhibitor of protein synthesis, alone or CHX + ganetespib (1 µM) for the indicated timepoints. As shown in Fig. 5a,b, ErbB2 protein levels were significantly decreased after 4 hours of CHX + ganetespib treatment as compared to CHX treatment alone in both cell lines. Overall, these data support ErbB2 as a client protein of HSP90 and indicate that the selective targeting of ErbB2 is a critical component of the anti-cancer mechanism of ganetespib in ErbB2^+^ breast cancer cells.

### Ganetespib preferentially inhibits ErbB2^+^ cancer cells

**Figure 6:**
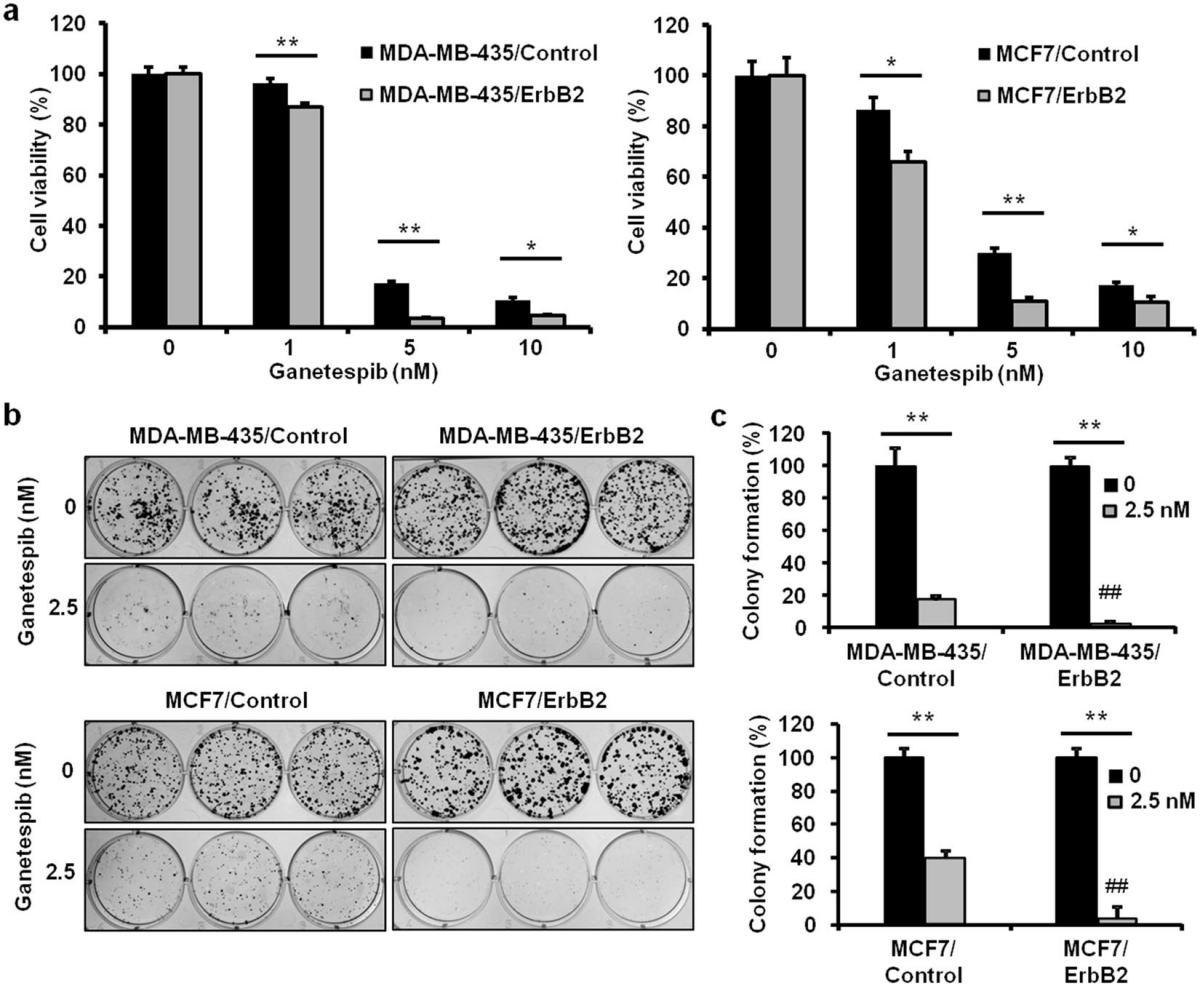
Ganetespib preferentially inhibits ErbB2^+^ cancer cells. (**a**) MDA-MB-435/Control, MDA-MB-435/ErbB2, MCF7/Control, and MCF7/ErbB2 cells were treated with ganetespib (0, 1, 5, or 10 nM) and for 5 days, followed by an MTT assay to assess cell viability. The average percentages of viable cells relative to the untreated control cells in each cell line are graphed. For the clonogenic assay, these cells were treated with ganetespib (0 or 2.5 nM) for 14 days. Then, colonies were stained with crystal violet and quantified. Representative images of the crystal violet-stained colonies are shown in (**b**) with the corresponding graphs in (**c**). All values are presented as the means ± S.E. (*P ≤ 0.05; **P ≤ 0.01 as indicated; ^##^*P* ≤ 0.01 as compared to the ganetespib-treated parental Control cells).

Since our findings indicate that ganetespib induces the degradation of ErbB2 in ErbB2^+^ breast cancer cells (Fig. 5), we aimed to determine whether ErbB2-overexpressing cancer cells are more sensitive to ganetespib than ErbB2-negative cells. To this end, we examined the selectivity of ganetespib-induced anti-proliferative effects in ErbB2^+^ cancer by overexpressing ErbB2 in two cancer cell lines that are typically ErbB2 deficient (MDA-MB-435 and MCF7) (Supplementary Fig. S1). Parental MDA-MB-435 and MCF7 cells (MDA-MB-435/Control and MCF7/Control) and isogenic ErbB2-overexpressing cells (MDA-MB-435/ErbB2 and MCF7/ErbB2) were treated with ganetespib (0, 1, 5, or 10 nM) for 5 days, followed by an MTT assay. As shown in Fig. 6a, ganetespib-mediated decreases in cell viability were more significant in the ErbB2-overexpressing cell lines as compared to the ErbB2-negative parental cells lines. Similarly, ErbB2 overexpression substantially amplified the inhibitory effects of ganetespib (2.5 nM for 14 days) on colony formation in both MDA-MB-435/ErbB2 and MCF7/ErbB2 cells as compared to MDA-MB-435/Control and MCF7/Control cells (Fig. 6b,c). To note, ectopic ErbB2 overexpression did not induce evident changes in HSP90 and HSP70 expression (Supplementary Fig. S1), suggesting that the increased sensitivity to ganetespib in ErbB2-overexpressing cells does not result from altered HSP90/HSP70 expression, but rather ganetespib-induced suppression of HSP90 activity leads to ErbB2 degradation. Together, these critical data demonstrate that ErbB2^+^ cancer cells are preferentially sensitive to the anti-cancer effects of ganetespib, particularly on cell proliferation, which indicate the fundamental role of ErbB2 in the targeted effects of ganetespib.

### Ganetespib significantly inhibits ErbB2-overexpressing mammary tumors *in vivo* in a syngeneic transplantation mouse model

**Figure 7:**
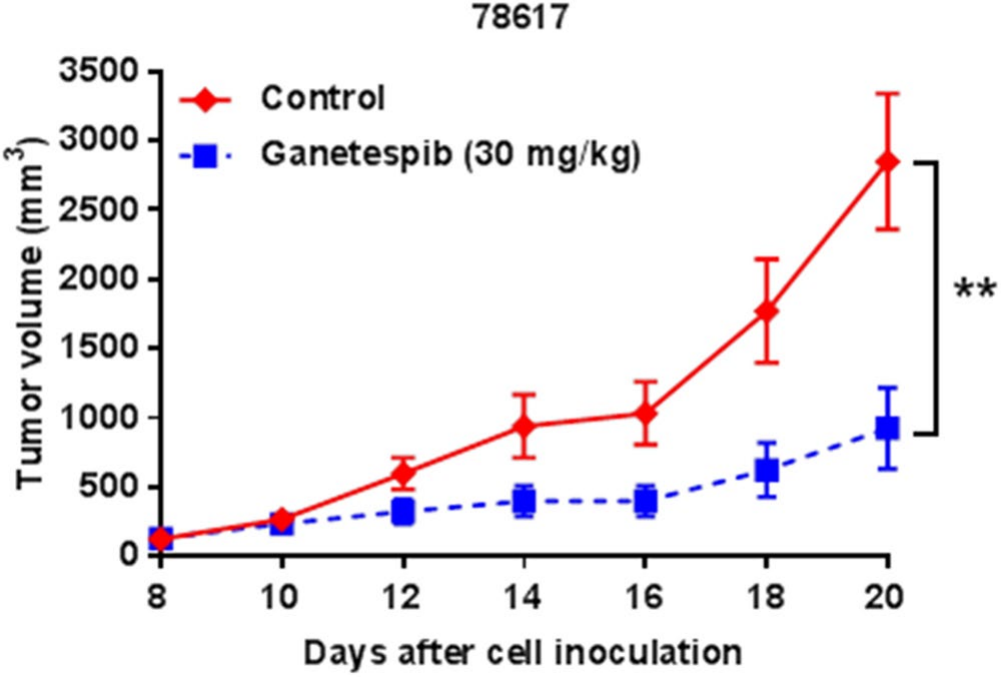
Ganetespib significantly inhibits ErbB2-overexpressing mammary tumors *in vivo* in a syngeneic transplantation mouse model. 10-week-old MMTV-ErbB2 mice (N = 6 mice per group) were subcutaneously injected with 78617 cells for syngeneic tumor growth. Once tumors were palpable 8 days after the initial tumor cell inoculations, ganetespib (30 mg/kg) was administered via i.p. injections every other day until the 20^th^ day after tumor cell inoculations. Tumor volumes were measured every 2 days. All values are presented as the means ± S.E. (**P ≤ 0.01).

To validate our *in vitro* findings that ErbB2-overexpressing cancer cells are highly sensitive to ganetespib, we tested the effects of ganetespib on ErbB2-overexpressing mammary tumor growth *in vivo*. To this end, ErbB2-overexpressing 78617 mammary tumor cells, which were derived from mammary tumors of MMTV-ErbB2 transgenic mice, were subcutaneously transplanted into syngeneic 10-week-old MMTV-ErbB2 mice. Upon palpable tumor formation, ganetespib (30 mg/kg) was administered via intraperitoneal injection every 2 days for 12 days. We found that ganetespib remarkably inhibited ErbB2-overexpressing tumor growth with an average final tumor volume of 922.8 ± 291.7 mm^3^ in the ganetespib-treated mice versus 2848.6 ± 489.6 mm^3^ in the control mice (Fig. 7). The significant ganetespib-induced tumor growth inhibition *in vivo* supports our *in vitro* data demonstrating the anti-cancer potential of ganetespib.

### Ganetespib potentiates the anti-cancer effects of lapatinib in ErbB2^+^ breast cancer cells

**Figure 8:**
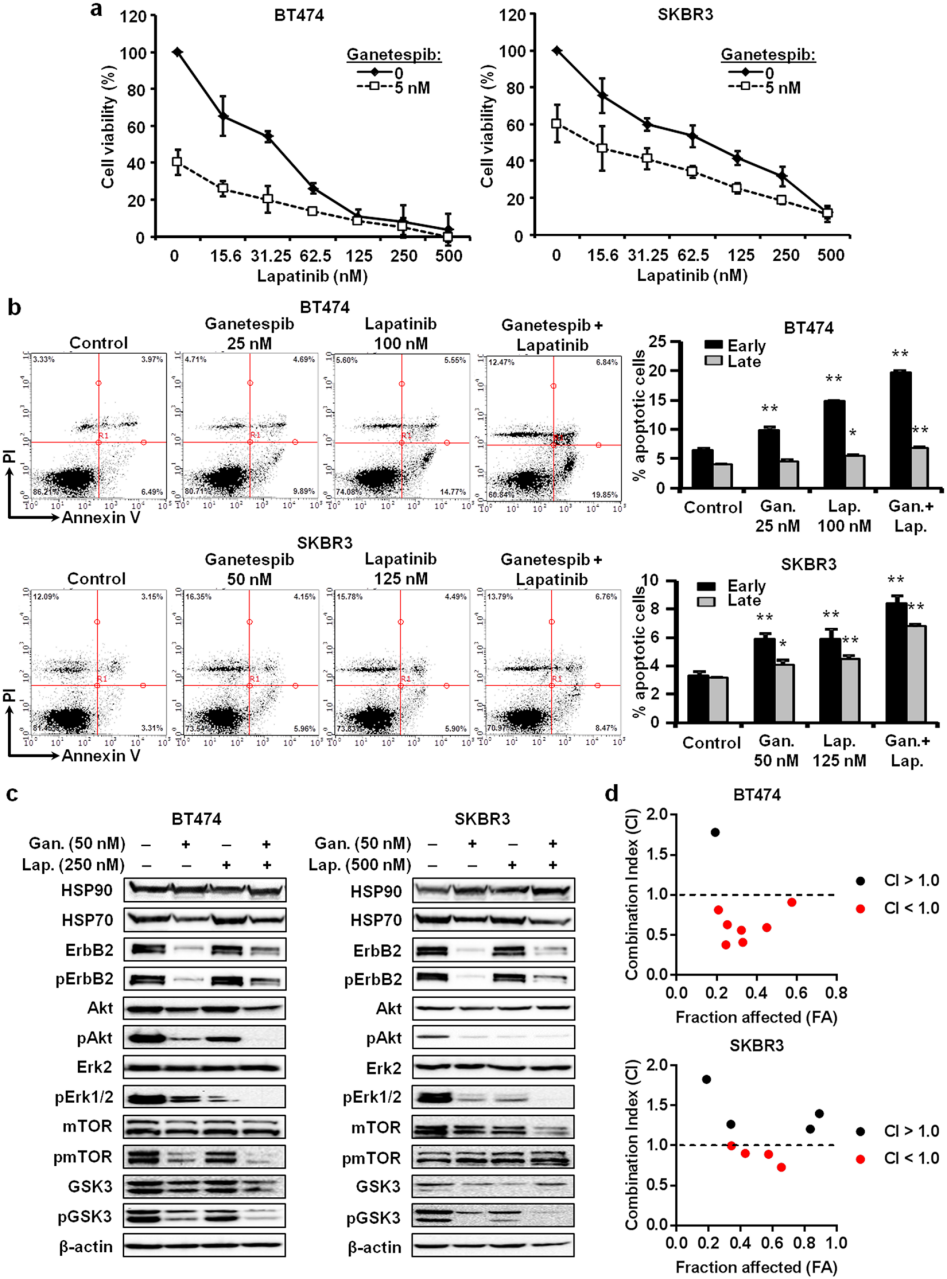
Ganetespib potentiates the anti-cancer effects of lapatinib in ErbB2^+^ breast cancer cells. (**a**) BT474 and SKBR3 cells were treated with ganetespib (0 or 5 nM) and lapatinib (0, 15.6, 31.25, 62.5, 125, 250, or 500 nM) for 5 days, followed by an MTT assay to assess cell viability. (**b**) BT474 and SKBR3 cells were treated with ganetespib alone (25 or 50 nM), lapatinib alone (100 or 125 nM), or ganetespib + lapatinib for 24 hours. Then, cells were Annexin V/PI-stained and analyzed with flow cytometry. The percentages of cells in early (Annexin V^+^/PI^−^) and late (Annexin V^+^/PI^+^) stages of apoptosis are graphed in the panels to the right of the representative dot plots. (**c**) BT474 cells were treated with ganetespib (50 nM) +/− lapatinib (250 nM) for 16 hours, and SKBR3 cells were treated ganetespib (50 nM) +/− lapatinib (500 nM) for 16 hours. Then, Western blot analysis was performed to detect the expression and activation/phosphorylation of the indicated markers. Cropped Western blot images are shown at the target molecular weights for the indicated markers. (**d**) BT474 and SKBR3 cells were treated with a series of increasing doses of ganetespib and lapatinib for 5 days. Then the viable fraction of each cell line (fraction affected; FA) was determined with an MTT assay. The Combination Index (CI) was calculated using CompuSyn software and graphed for each cell line. Dose combinations with a CI < 1.0 are highlighted in red. All values are presented as the means ± S.E. (*P ≤ 0.05; **P ≤ 0.01 as compared to the corresponding untreated controls).

The combination of ErbB2-targeting therapeutics, like lapatinib, with other anti-cancer agents has been used as therapeutic strategy in the clinic to increase drug potency and improve patient responses. In particular, HSP90 inhibitors in combination with ErbB2-targeting therapeutics have shown promising anti-cancer efficacy^42,43^. As such, since lapatinib and ganetespib target ErbB2 via different mechanisms, we proposed that the combinational treatment of lapatinib + ganetespib may have synergistic inhibitory effects on ErbB2^+^ breast cancer cells. To this end, BT474 and SKBR3 cells were treated with increasing concentrations of lapatinib (0–500 nM) with or without ganetespib (5 nM) for 5 days and cell viability was assessed with an MTT assay. As shown in Fig. 8a, BT474 and SKBR3 cell viability was drastically reduced in lapatinib + ganetespib-treated cells as compared to lapatinib treatment or ganetespib treatment alone, indicating synergistic inhibitory effects on ErbB2^+^ breast cancer cell viability.

In addition to the anti-proliferative actions, previous studies report that lapatinib and ganetespib can individually attenuate cell survival in various cancer models^27,28,44,45^. However, the combined effects of lapatinib + ganetespib on apoptosis have also not been reported. Therefore, we analyzed Annexin V/PI staining to determine the effects of lapatinib and/or ganetespib on apoptosis induction in BT474 and SKBR3 cells. Our flow cytometry data indicated that ganetespib alone and lapatinib alone strikingly induced early stage (Annexin V^+^/PI^−^) apoptosis, with less evident effects on late stage (Annexin V^+^/PI^+^) apoptosis in both cell lines (Fig. 8b). Notably, the combination treatment of ganetespib + lapatinib demonstrated significant synergistic effects on the induction of both early and late stages of apoptosis.

Given synergistic effects of ganetespib + lapatinib on cell growth inhibition and apoptosis induction, and the regulatory role that RTK signaling plays in these cellular responses, we further explored whether the combination treatment of ganetespib and lapatinib enhanced RTK signaling inhibition in ErbB2^+^ breast cancer cells. Indeed, we demonstrated that ganetespib (50 nM) and/or lapatinib (250–500 nM) treatment decreased the activation of ErbB2 and key downstream targets of the RTK pathway in BT474 and SKBR3 cells as compared to the untreated control cells (Fig. 8c). In particular, the combination treatment significantly amplified the inhibition of Akt, Erk1/2, mTOR, and GSK3 activation. Ganetespib also did not significantly alter HSP90 and HSP70 expression, which suggests that the decreases in ErbB2, Akt, mTOR, and GSK3 protein expression result from ganetespib-induced inhibition of HSP90-associated chaperone function.

To further confirm the synergistic relationship of ganetespib + lapatinib, we performed a series of dose-effect analyses of the combined effects of ganetespib + lapatinib on BT474 and SKBR3 cells. As shown in the Combination Index (CI) plot of appropriate ganetespib + lapatinib dose ranges in Fig. 8d, synergistic effects were evident at effect levels (fraction affected; FA) greater than 20% and 35% in BT474 and SKBR3 cells, respectively, suggesting that the synergy between ganetespib and lapatinib was more pronounced in BT474 cells. These results are consistent with our previous report showing the combination treatment of ganetespib and lapatinib enhances RTK signaling inhibition in gastric cancer cells^28^, which further indicate the potential application of ganetespib + lapatinib as a synergistic combinational therapy for ErbB2^+^ breast cancer.

## Discussion

In the present study, we investigated the effects of ganetespib on ErbB2^+^ breast cancer cells with mechanistic insight. Our results demonstrated that ganetespib stimulates striking growth inhibition and apoptosis in ErbB2^+^ breast cancer cells. Molecular analyses indicated that ganetespib acts on multiple key regulators of cell cycle progression and apoptosis. *In vivo* data provided critical evidence that ganetespib inhibits ErbB2-overexpressing mammary tumor growth in MMTV-ErbB2 mice inoculated with syngeneic mammary tumor cells. We also showed that ErbB2-overexpressing breast cancer cells are more sensitive to ganetespib as compared to ErbB2-deficient parental breast cancer cells. Our data support targeting HSP90, via ganetespib, as a promising therapeutic strategy for ErbB2^+^ breast cancer.

HSP90 plays an essential role in the folding, assembly, and proteolytic turnover of various client proteins^7,8^. In particular, ErbB2 is one of the most sensitive HSP90 client proteins^12,13^. As such, targeting ErbB2^+^ breast cancer with HSP90 inhibitors, such as ansamycin and its derivatives, has been tested in previous studies^9,23,46,47^. However, toxicities and efficacy issues associated with first generation HSP90 inhibitors have hindered the further clinical application of HSP90 inhibitors. Ganetespib, as part of a new generation of HSP90 inhibitors with an improved safety profile, is emerging as a promising anti-cancer agent that is being tested in clinical trials^22–25^. Nevertheless, the effects of ganetespib on ErbB2^+^ breast cancer, and the cellular targets downstream of ganetespib-mediated HSP90 inhibition remain unclear. Our experiments showed that ganetespib potently inhibits ErbB2^+^ BT474 and SKBR3 breast cancer cells, as indicated by MTT and clonogenic assays (Fig. 1). Although G0/G1 phase arrest was cell line-specific in our study, cell cycle analyses indicated that ganetespib induces G2/M phase arrest in BT474 and SKBR3 cells (Fig. 2), which is consistent with previous reports from our lab and others showing that ganetespib induced G2/M phase arrest in gastric cancer cells^27,28^. Importantly, we found that ganetespib-induced cell cycle arrest was associated with the inhibition of Cyclin D1 and CDK1 and upregulation of Cyclin B1 and p27. The data suggest that the differential regulation of Cyclins and CDKs is a critical mechanism of ganetespib-mediated HSP90 inhibition and downstream cellular responses. Previous reports suggest that the downregulation of Cyclin D1 and upregulation of p27 are commonly associated with ErbB2 signaling inhibition^48^. In contrast, the upregulation of Cyclin B1 appears to be more specific to ganetespib-induced cell cycle regulation^28,30^. The mechanism of ganetespib-mediated regulation of Cyclin B1 is an intriguing question to be addressed.

Our results showed that, in addition to cell cycle arrest, ganetespib also induces significant apoptosis in ErbB2^+^ breast cancer cells, as indicated by the Annexin V/PI assay and PARP cleavage (Fig. 3). The analysis of caspase cleavage/activation indicated that in addition to caspase 3 cleavage, both caspase 8 and 9 cleavage was induced by ganetespib, even at low doses. Caspase 3 is an effector caspase, while caspase 8 and 9 are associated with death receptor and mitochondrial apoptotic pathways, respectively. Moreover, we also showed that ganetespib significantly downregulated Bcl-2, a master pro-survival regulator. Our results indicate that ganetespib-induced apoptosis involves both death receptor and mitochondrial pathways and Bcl-2 regulation, suggesting that ganetespib acts on multiple levels of the apoptotic cascade.

The activation of RTK signaling pathways is a driving force of ErbB2^+^ breast cancer cell proliferation and survival. Our analysis of key regulators downstream of ErbB2 indicated that ganetespib strikingly inhibited the activation/phosphorylation of ErbB2, Akt, Erk1/2, Src, mTOR, Bad, and GSK3 (Fig. 4). In contrast to most ErbB2-targeting agents, which mainly inhibit the phosphorylation of RTK signaling markers, the notable feature of ganetespib was the remarkable downregulation of ErbB2, Akt, Src, and GSK3 total protein expression, in addition to decreased phosphorylation, which suggests the involvement of protein degradation as a result of HSP90-associated chaperone function. Although previous reports indicate that HSP90 client proteins include ErbB2, Akt, Src, and GSK3^12,13,21,40^, results from this study underscore the multi-level actions of ganetespib on RTK signaling in ErbB2^+^ breast cancer cells.

To understand the specific interactions between ganetespib/HSP90 and ErbB2, we particularly examined the effects of ganetespib on ErbB2 protein stability and its effects on cancer cells with different ErbB2 statuses. Consistent with previous reports, ganetespib-induced ErbB2 degradation was significant in the presence of CHX (Fig. 5). This result validates ganetespib as a potential ErbB2-targeting agent and partially addresses the significant inhibition of RTK signaling that we observed. Importantly, to further evaluate the impact of ganetespib-mediated ErbB2-targeting function, we compared the cellular sensitivity of paired isogenic cancer cell lines with/without ErbB2 overexpression. Our results demonstrated that ErbB2-overexpressing cancer cells (MDA-MB-435/ErbB2 and MCF7/ErbB2) were more sensitive to ganetespib than the parental cells (MDA-MB-435/Control and MCF7/Control), as indicated by both MTT and clonogenic assays (Fig. 6). These results are of imperative significance because it suggests the preferred sensitivity of ErbB2-overexpressing cancer cells to ganetespib, which provides key preclinical evidence that supports the rationale for the application of ganetespib in ErbB2^+^ breast cancer.

Several ErbB2-targeting therapeutics, such as trastuzumab and lapatinib, have been used in clinical oncology with encouraging outcomes. Combining these therapeutics with other drugs is a major strategy to improve clinical efficacy and overcome drug resistance. In addition to its use as a monotherapy, ganetespib could be used in combination with existing ErbB2-targeting agents to enhance the potency or clinical effects. As such, ganetespib is being tested in combination with paclitaxel and trastuzumab in Phase I clinical trials^38^. This recent Phase I clinical trial by Jhaveri *et al*.^38^ not only provides a strong foundation with clinical relevancy for our current study focusing on the anti-cancer effects and mechanisms of ganetespib specifically in the ErbB2^+^ breast cancer subtype, but also fundamentally supports the concept that novel treatment strategies combining ganetespib with ErbB2-targeting therapeutics may improve clinical responses in ErbB2^+^ breast cancer patients. Given that ganetespib was well-tolerated in ErbB2^+^ breast cancer patients^38^ and that lapatinib, a dual inhibitor of EGFR and ErbB2, has been approved for the clinical treatment of metastatic ErbB2^+^ breast cancer^49^, we extended our study to examine whether ganetespib can enhance lapatinib-mediated inhibition of ErbB2^+^ breast cancer cells. This combination strategy is promising given that ganetespib and lapatinib target ErbB2 via different mechanisms, particularly HSP90 inhibition-mediated ErbB2/client protein degradation and direct tyrosine kinase inhibition, respectively. Our results demonstrated that ganetespib significantly potentiates lapatinib-induced growth inhibition and apoptosis (Fig. 8). Molecular analysis of RTK signaling in BT474 and SKBR3 ErbB2^+^ breast cancer cells indicated that the combination of lapatinib with low-dose ganetespib (50 nM) enhances the inhibition of ErbB2, Akt, Erk1/2, mTOR, and GSK3 activation/phosphorylation. These data demonstrating the synergistic effects of ganetespib-induced degradation of HSP90 client proteins and lapatinib-induced inhibition of apical RTK activity on various levels of the RTK pathways support potential clinical trials testing the combined treatment regimen of ganetespib + lapatinib in ErbB2^+^ breast cancer patients.

In this study, we demonstrated the potent anti-cancer effects of ganetespib on ErbB2^+^ breast cancer cells *in vitro* and *in vivo*. The underlying mechanisms include the induction of cell cycle arrest and apoptosis, which resulted from the inhibition of RTK signaling, differential regulation of Cyclins/CDKs, and modulation of apoptotic machinery. Molecular dissection of ganetespib-mediated signaling in these pathways indicate that ganetespib can act on multiple targets at different levels of the signaling cascades regulating RTK pathways, cell cycle progression, and the balance between cell death and survival. In addition to ErbB2 targeting-induced inhibition of kinase phosphorylation/activation, ganetespib-mediated downregulation of the protein levels of downstream effectors, including Akt, Src, and GSK3, also contributes to effective RTK signaling inhibition. Ganetespib-induced G2/M phase cell cycle arrest, and concurrent CDK1 downregulation and Cyclin B1 upregulation may involve regulatory mechanisms beyond ErbB2 inhibition alone, which warrants further investigation. Although ErbB2 signaling is involved in apoptosis regulation, such as via the inhibition of Bad phosphorylation, sensitive caspase 8 cleavage/activation and Bcl-2 downregulation in ganetespib-treated cells suggests that ganetespib induces multi-faceted regulation of the apoptotic cascade. The integration of our results suggests that the simultaneous regulation of multiple pathways and preferential sensitivity in ErbB2^+^ breast cancer cells makes ganetespib a promising therapeutic agent for refractory ErbB2^+^ breast cancers. Our findings provide fundamental evidence supporting future preclinical and clinical testing of ganetespib in ErbB2^+^ breast cancer.

In conclusion, our data demonstrate that ganetespib is a potent therapeutic agent for ErbB2^+^ breast cancer cells. Our molecular analyses indicate that ganetespib, in addition to targeting ErbB2, may act on multiple intracellular targets to regulate RTK signaling, cell cycle progression, and apoptosis. In particular, we demonstrated that ErbB2-overexpressing cancer cells are preferentially responsive to ganetespib, and that ganetespib significantly potentiates lapatinib-mediated anti-cancer effects in ErbB2^+^ breast cancer cells. Together, our compelling data provide a strong foundation for continued investigation regarding the clinical impact of ganetespib as a promising therapeutic option for patients with ErbB2^+^ breast cancer.

## Materials and Methods

### Chemical and antibodies

Ganetespib was purchased from Medkoo Biosciences, Inc. (Chapel Hill, NC). Lapatinib was purchased from LC Laboratories (Woburn, MA). Cycloheximide was purchased from Sigma-Aldrich (St. Louis, MO). Primary antibodies specific to cleaved PARP, cleaved caspase 3, cleaved caspase 8, cleaved caspase 9, Bcl-2, ErbB2, phospho-ErbB2 (pErbB2), Akt, pAkt, Erk2, pErk1/2, mTOR, pmTOR, Cyclin B1, Src, pSrc, Bad, pBad, GSK3, and pGSK3 were purchased from Cell Signaling Technology (Danvers, MA). Primary antibodies against CDK1, Cyclin D1, p27, caspase 8, caspase 9, HSP90, HSP70, and β-actin were purchased from Santa Cruz Biotechnology (Santa Cruz, CA). Secondary mouse and rabbit antibodies were purchased from Thermo Scientific (Rockford, IL).

### Cell culture

BT474, SKBR3, MCF7, and MDA-MB-435 cells were purchased from the American Type Culture Collection (ATCC; Manassas, VA). MCF-7/ErbB2 and MDA-MB-435/ErbB2 (MDA-MB-435/EB1) cell lines were kindly provided by Dr. Christopher C. Benz (Buck Institute for Research on Aging) and Dr. Dihua Yu (MD Anderson Cancer Center)^50^, respectively. Cells were maintained in Dulbecco’s Modified Eagle’s-F12 (DMEM/F12) medium (Life Technologies; Carlsbad, CA) with 10% fetal bovine serum, 100 μg/mL penicillin, and 100 μg/mL streptomycin at 37 °C in a humidified atmosphere containing 5% CO_2_. Cells were passaged with 0.25% trypsin-EDTA at ~80% confluency.

### Cell viability assay

For the MTT cell viability assay, 1 × 10^3^ cells/well were seeded in a 96-well plate and allowed to adhere overnight. The next day, cells were treated with the indicated concentrations of ganetespib and/or lapatinib for 5 days. Following drug treatments, MTT reagent was added to each well for 3 hours to allow for crystal formation. The media was aspirated and the crystals were dissolved in DMSO by shaking on rocker for 45 minutes. Absorbance at 570 nm was measured using a BioTek SynergyMx plate reader (BioTek; Winooski, VT). The percentage of viable ganetespib- and/or lapatinib-treated cells was calculated relative to the control vehicle-treated cells. All experiments were performed in at least triplicate.

### Clonogenic assay

To measure the colony-forming ability, BT474, SKBR3, MDA-MB-435/Control, MDA-MB-435/ErbB2, MCF7/Control, and MCF7/ErbB2 cells were seeded at 1 × 10^3^ cells/plate in 60 mm dishes and grown overnight. The cells were then treated with indicated concentrations of ganetespib for 21 days (BT474 cells) or 14 days (SKBR3, MDA-MB-435/Control, MDA-MB-435/ErbB2, MCF7/Control, and MCF7/ErbB2 cells), followed by staining with 0.5% crystal violet (1:1 methanol:H_2_O) for 30 minutes at room temperature. Images of the stained colonies were taken with a digital camera mounted on a Nikon C-LEDS microscope and quantified using AlphaView software (ProteinSimple; San Jose, CA).

### Cell cycle analysis

BT474 (1 × 10^6^ cells/plate) and SKBR3 (1 × 10^6^ cells/plate) cells were seeded in 60 mm dishes. After growing for 24 hours, cells were treated with the indicated concentrations of ganetespib for 24 hours. Cells were trypsinized, harvested, and fixed overnight in 70% (v/v) ethanol at −20 °C. After incubation, cells were washed and centrifuged at 1500 rpm for 5 minutes and the cell pellets were resuspended in 33 μg/mL PI containing 1 mg/mL RNase solution (Sigma-Aldrich) and incubated at 37 °C for 45 minutes. Cells were then filtered through 40 μm nylon mesh. Cell cycle progression was analyzed with a Guava EasyCyte 8 Flow Cytometer (Millipore; Billerica, MA) and cell cycle distribution was calculated using ModFit 3.0 software (Verity Software House; Topsham, ME).

### Western blot analysis

For Western blot analyses, cells were treated with the indicated concentrations of ganetespib and/or lapatinib for 16 hours. Then, the cells were collected and lysed using NP40-based lysis buffer. The protein concentration of each sample was determined using a BCA assay (Thermo Scientific). Equal amounts of protein (50 μg) from each sample were loaded for SDS-PAGE and the separated proteins were transferred to nitrocellulose membranes. The membranes were washed in TBST and blocked in 5% non-fat milk for 2 hours. Then, the membranes were washed in TBST and incubated overnight in the indicated primary antibodies at 4 °C. The membranes were washed again in TBST, followed by incubation in the appropriate species-specific secondary antibody for 1 hour at room temperature. The protein signals were enhanced with ECL solution (Thermo Scientific) and the bands were imaged using a FluorChemE imager (Cell Biosciences; Santa Clara, CA).

### Apoptosis assay

Cells (5 × 10^5^ cells/plate) were seeded in 60 mm dishes overnight, followed by treatment with the indicated concentrations of ganetespib and/or lapatinib for 24 hours. Then, cells were collected, washed, and stained using an Annexin-V-FLUOS staining kit (Sigma-Aldrich) according to the manufacturer’s instructions. The samples were analyzed using a Guava EasyCyte 8 Flow Cytometer and the distribution of apoptotic cells was calculated using ModFit software.

### CHX-based ErbB2 protein stability assay

Cells (5 × 10^5^ cells/plate) were seeded in 60 mm dishes. The next day, cells were treated with 100 μg/mL of CHX alone or with ganetespib (1 µM) and harvested at the indicated time points. Proteins were extracted and analyzed by Western blotting to assess ErbB2 protein stability. Protein band intensities were semi-quantitatively analyzed by densitometry using ImageJ software (National Institutes of Health; Bethesda, MD). ErbB2 protein band intensities for each treatment condition were normalized to corresponding β-actin protein band intensities. The protein degradation linear regression curve was generated by plotting band intensity ratios as a function of the duration of CHX exposure.

### Animals and treatments

Female FVB/N-Tg MMTV-neu/ErbB2 (MMTV-ErbB2) transgenic mice were purchased from Jackson Laboratories (Bar Harbor, ME). All animal procedures were approved by the Institutional Animal Care and Use Committee (IACUC) of the North Carolina Research Campus (NCRC) and were carried out according to relevant guidelines and regulations. For the syngeneic tumor cell inoculation, approximately 5 × 10^5^ 78617 cells in 1:1 matrigel:RPMI 1640 medium were injected subcutaneously into the flanks of 10-week-old MMTV-ErbB2 mice (N = 6 mice per group). The syngeneic tumor size was monitored every 2 days throughout the experiment and tumor volume was calculated as: tumor volume (mm^3^) = (width^2^ × length)/2. Upon the first appearance of palpable tumors at day 8 after the tumor cell inoculations, the mice were randomly divided into equal groups for vehicle and ganetespib (30 mg/kg) treatments. The treatments were administered every 2 days via intraperitoneal (i.p.) injection. At 20 days after the initial tumor cell inoculations, the mice were euthanized and tumors were collected and imaged.

### Dose-effect and synergistic (CI) analyses

To determine the pharmacological drug interactions between ganetespib and lapatinib, cells were treated for 5 days with a series of drug combinations ranging from 2–100 nM ganetespib and 6–600 nM lapatinib, followed by an MTT assay, as performed according the procedures described above. Then, CI values were calculated using the Chou-Talalay method with CompuSyn software (ComboSyn, Inc.; Paramus, NJ). CI < 1, CI = 1, and CI > 1 indicate synergistic, additive, and antagonistic effects, respectively^51^.

### Statistical analysis

GraphPad Prism software (La Jolla, CA) was used for the statistical analyses. A Student’s t-test or one-way analysis of variance (ANOVA) was used to determine the significant differences between groups. A *P*-value of ≤ 0.05 (*P* ≤ 0.05) was chosen to indicate significance.

## Electronic supplementary material

Supplementary Information
