# Supplementary material for: Ganetespib targets multiple levels of the receptor tyrosine kinase signaling cascade and preferentially inhibits ErbB2-overexpressing breast cancer cells
Source: Sci Rep. 2018 May 1;8:6829. doi: 10.1038/s41598-018-25284-0 (PMC5931511; doi:10.1038/s41598-018-25284-0)

Ganetespib targets multiple levels of the receptor tyrosine kinase signaling cascade and preferentially inhibits ErbB2-overexpressing breast cancer cells

Harry Lee[1], Nipun Saini[1], Erin W. Howard[1], Amanda B. Parris[1], Zhikun Ma[1], Qingxia Zhao[1], Ming Zhao[1], Bolin Liu[2], Susan M. Edgerton[2], Ann D. Thor[2], Xiaohe Yang[1]*

[1]Julius L. Chambers Biomedical/Biotechnology Research Institute, Department of Biological and Biomedical Sciences, North Carolina Central University, North Carolina Research Campus, Kannapolis, North Carolina

[2]Department of Pathology, School of Medicine, University of Colorado Anschutz Medical Campus, Aurora, Colorado

*xyang@nccu.edu

Supplementary Figure Legends

Supplementary Figure S1: Endogenous protein expression of HSP90 and HSP70 is not altered in MDA-MB-435 and MCF7 cells with different ErbB2 status. Protein expression and activation/phosphorylation of the indicated markers were analyzed in untreated MDA-MB-435/Control, MDA-MB-435/ErbB2, MCF7/Control, and MCF7/ErbB2 cells. Cropped Western blot images are shown with "C" indicating the control cell lines and "+" indicating the cell lines with induced ErbB2 overexpression.

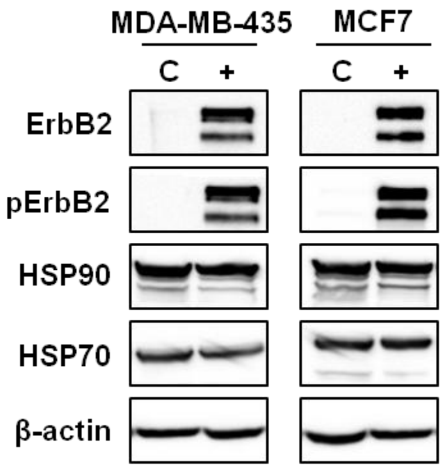

Supplement: Supplementary file 1 — Supplementary Information [file 41598_2018_25284_MOESM1_ESM.pdf]
